# Unmet healthcare needs in homeless women with children in the Greater Paris area in France

**DOI:** 10.1371/journal.pone.0184138

**Published:** 2017-09-06

**Authors:** Cécile Vuillermoz, Stéphanie Vandentorren, Ruben Brondeel, Pierre Chauvin

**Affiliations:** 1 Sorbonne Universités, UPMC Univ Paris 06, INSERM, Institut Pierre Louis d’épidémiologie et de Santé Publique (IPLESP UMRS 1136), Department of Social Epidemiology, Paris, France; 2 Direction des régions, Santé publique France, Saint Maurice, France; 3 Sorbonne Universités, UPMC Univ Paris 06, INSERM, Institut Pierre Louis d’épidémiologie et de Santé Publique (IPLESP UMRS 1136), Nemesis team, Paris, France; International University of Health and Welfare School of Medicine, JAPAN

## Abstract

**Background:**

Despite their poor health status, homeless women encounter many barriers to care. The objectives of our study were to estimate the prevalence of unmet healthcare needs in homeless women and to analyse associated relationships with the following factors: financial and spatial access to care, housing history, migration status, healthcare utilisation, victimization history, caring for children, social network and self-perceived health status.

**Methods:**

We used data from 656 homeless women interviewed during the ENFAMS representative survey of sheltered homeless families, conducted in the Paris region in 2013. Structural equation models (SEM) were used to estimate the impact of various factors on homeless women’s unmet healthcare needs.

**Results:**

Among those interviewed, 25.1% (_95%_CI[21.3–29.0]) had at least one unmet healthcare need over the previous year. Most had given up on visiting general practitioners and medical specialists. No association with factors related to financial access or to health insurance status was found. However, food insecurity, poor spatial health access and poor self-perceived health were associated with unmet healthcare needs. Self-perceived health appeared to be affected by victimization and depression.

**Discussion:**

The lower prevalence of unmet healthcare needs in homeless women compared with women in stable housing situations suggests that homeless women have lower needs perceptions and/or lower expectations of the healthcare system. This hypothesis is supported by the results from SEM. Strategies to provide better access to care for this population should not only focus on financial interventions but also more broadly on spatial healthcare access, cultural norms, and perceptions of health. Reducing their unmet needs and improving their access to healthcare and prevention must include an improvement in their living, financial and housing conditions.

## Introduction

The homeless population in Europe has continued to increase in the past 10 years. It is mainly composed of migrants, young people, women, and families [1]. Homelessness increased by 50% in France between 2001 and 2012, reaching an estimated 141,500 people [2]. With a 300% increase in the number of homeless families using accommodation services, families represented the fastest growing segment in the homeless population in the greater Paris area from 1999 to 2011 [3,4]. Since these families were mainly composed of single mothers with children, women became a significantly larger subpopulation within the homeless population [5].

Previous studies on health and on access to healthcare in the homeless women population, mainly conducted in North America, revealed that individuals’ physical and mental health status was a major cause of concern [6]. Homeless people, in particular young people and women, have a higher risk of dying prematurely than the general population [1]. In particular, they are more likely to have mental diseases, chronic diseases, sexually transmitted infections and gynaecological problems [7].

Homeless women encounter many barriers to care. In the US, some studies have suggested that poor resources and a lack of health insurance are the main barriers to accessing homeless healthcare [8,9]. Other studies have shown that homeless people with health insurance face substantial non-financial barriers such as competing needs, a small social network, housing instability (due to frequent moves from one shelter to another), a lack of knowledge about places providing care, a lack of transportation and/or low daily mobility, long healthcare waiting times, and perceived discrimination [10–19]. Moreover, compared with homeless men, women face an additional problem: services targeting the homeless population have mainly been designed for men and may not be suited to women’s needs [13]. In France, a context where US results may be not transferable, very few recent studies have been conducted examining healthcare utilization by homeless women [20]. Nonetheless, they confirm that this population suffers from poor physical and mental health. However, these studies did not document homeless women’s unmet healthcare needs.

Healthcare utilization is usually evaluated by the number of times a person utilizes the healthcare system, in particular the number of consultations with general practitioners, specialists and other healthcare services (emergency units, dental care, glasses/contact lenses). The measure of unmet healthcare needs is used as an indicator of non-access to healthcare in many European and American surveys [21–25]. An unmet need is a situation where an individual wants to use healthcare services but is faced with constraints related to healthcare accessibility. In France, this indicator is regularly monitored in the general population and its time trend is used as an indicator for public policy makers, e.g. for the annual vote of the Social Security Financing Act by the Parliament [26].

The objectives of our study were to estimate the prevalence of unmet healthcare needs in homeless women and to describe associated relationships with a set of individual characteristics selected from the scientific literature.

## Materials and methods

### Study sample

This study was based on data collected during ENFAMS, the first survey in France to focus on homeless families [27]. An eligible family was defined as comprising at least one parent (>18-year old) with at least one child younger than 13 years, sheltered in the Greater Paris area in social hotels, emergency centres, centres for asylum-seekers and long-term rehabilitation centres, speaking one of the 17 languages of the survey and able to provide written consent to participate.

The sampling process has been detailed elsewhere [27,28]. The sampling design included three levels of random sampling: shelters (which were randomly selected among an exhaustive list of all services in the Paris region), families (which were randomly selected in each selected service; either the single parent or one of the two parents was interviewed: in 95.4% of the cases this was the mother), and one child from every family.

The final sample of ENFAMS survey consisted of 801 families interviewed face-to-face, with the survey questionnaire administered in 17 languages by a team of bilingual (French/other language) interviewers.

The parent questionnaire collected demographics and socio-economic data, housing history since the first episode of homelessness, social relationships and social support, health conditions and self-reported health using the Health Perceptions Questionnaire [29] and healthcare utilization. The food insecurity was assessed using the Household Food Security Survey Measure [30]. It also focused on mental health using the World Health Organization CIDI-S [31,32] to measure depression and the MINI-S [33] to assess post-traumatic stress disorders among parents. The questionnaire has been more described in details elsewhere [27].

In this study, among the total number of women interviewed (N = 764), we excluded those who did not answer the question about unmet healthcare needs (N = 3, 0.39%) and those who arrived in France during the previous year (N = 105, 13.8%). Accordingly, our study sample comprised 656 homeless women.

Participants to the ENFAMS survey provide their written informed consent to participate to this study and the consent for their child who is involved in the study. The Review board “Comité de Protection des Personnes d’Île-de-France” approved the study protocol as well as this consent procedure, as well as the CNIL (the board enforcing laws on data protection) and the CCTIRS (a government committee consulted on issues dealing with information concerning health research).

### Hypothesized measurement model

**Fig 1:**
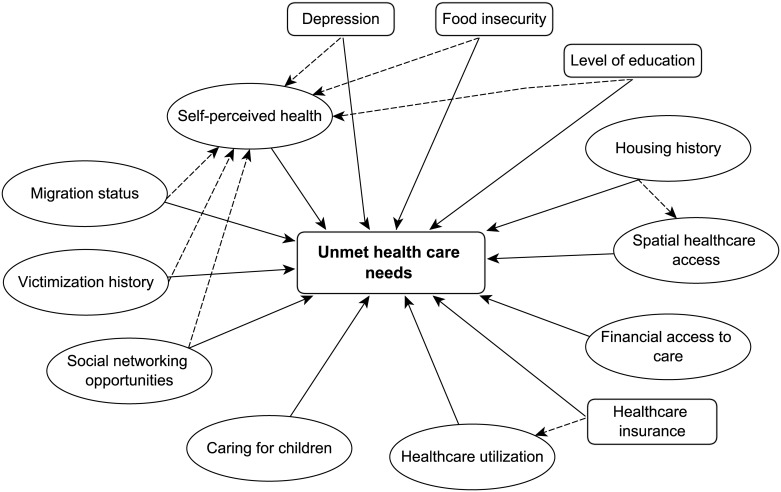
Hypothesized model of relationships between latent constructs of the unmet healthcare needs of homeless women and their characteristics. Ellipses: latent variables; boxes: observed variables.

Quantitative studies on factors associated with healthcare utilization usually use classic regression methods. However, as we wished to study the complex relationships between unmet healthcare needs and various associated factors found in previous studies, we deemed this approach inappropriate (due to difficulties establishing links between two or more concepts in a theoretical model). We felt that Structural Equation Models (SEM) were more suited [34,35]. Concepts (latent variables) not directly observable but constructed with observed variables (so-called ‘indicators’) could be taken into account, and the validity and reliability of these concepts could be tested [36,37]. We constructed a model based on a hypothesis-driven approach, as found in the literature. Given that unmet healthcare needs are based on individuals’ subjective needs, people must be able (i) to interpret their symptoms (and/or their perceived health status) as a matter for medical intervention, for which the use of a healthcare service may be useful; and (ii) to perceive this service as inaccessible for one reason or another. The following respective hypotheses were tested in our model (see Fig 1):

1. Perceived health status and/or interpretation of symptoms vary from one person to another [15], according to their health norms [38], their history of adverse life events [19,39,40], their mental health [19,41], their history of homelessness [42], their social network [43], their health literacy [44,45], and their past experiences and present expectations regarding sickness and care [13,44]. Therefore, we investigated the relationship between unmet healthcare needs and six constructed concepts: housing history, migration status, victimization history, social networking opportunities, healthcare utilisation (which we supposed was impacted by health insurance status), and perceived health.
2. Individuals must perceive this service as inaccessible because of financial and/or non-financial barriers, that is to say, not only lack of financial resources and lack of health insurance [8,15,17,19], but also transport, distance from the shelter to healthcare location, time needed, availability of services, cost, and competition between needs (food, clothing, children care) [10,14–16,18,42]. We investigated a series of relationships between unmet healthcare needs and a) three constructed concepts: financial access to care, spatial healthcare access (which we supposed was impacted by housing history) and caring for children, and b) three observed variables: level of education, health insurance status, and food insecurity status.

Since perceived health is also a subjective concept, we hypothesised its relationship with depression, migration status, victimization history, social networking opportunities, food insecurity and level of education [15,46]. Therefore, the potential impact of these 6 dimensions on unmet healthcare needs was tested both directly and indirectly, through perceived health.

### Variables used

#### Outcome

The following question was used to examine unmet healthcare needs: “In the last 12 months, have you given up visiting general practitioners or given up other medical care for any reason?” The others medical cares could be consultations, visits or treatment by a medical specialist, dental prostheses, dental care, glasses and contact lenses, lab tests, imaging, physical therapy, rehabilitation sessions, drugs delivery or other care.

#### Latent variables

Financial access to care was constructed from three indicators: (i) being unemployed (yes/no); (ii) having a monthly household income below the median value in the study population (*i*.*e*. 211 €/CU–where CU are consumption units counted as follows: the first adult of the household counts for 1 CU; other persons aged 14 and over 0.5 CU; children under 14 0.3 CU) (reply: yes/no); (iii) not having received social benefits during the previous year (including unemployment benefit, minimum income benefit, adult disability allowance, family allowances including all types of financial assistance to the poor, to the unemployed, to the disabled, to asylum-seekers and to parents etc.) (yes/no).

Migration status was constructed from four indicators: (i) being born in France or not, (ii) the time lived/spent in France (less or more than a quarter of one’s life), (iii) having difficulties with the French language (yes/no), and (iv) residence status at the time of the survey (legally resident or undocumented).

Housing history was constructed from three variables: (i) the duration of homelessness (more or less than 24 months), (ii) the number of moves per year since the first episode of homelessness (more or less than 4 moves per year), (iii) the duration spent in the current shelter (more or less than one year).

Spatial healthcare access was constructed from three indicators: (i) having difficulties using public transportation (yes/no); (ii) satisfaction with public transportation availability, and (iii) satisfaction with the supply of healthcare providers in neighbourhood where the shelter was located (both (ii) and (iii) had 4 possible answers to indicate satisfaction level–totally agree, somewhat agree, somewhat disagree, totally disagree).

Healthcare utilisation was constructed from three indicators: (i) not having consulted a physician during the previous year (yes/no), (ii) not having a regular gynaecological follow-up (yes/no), (iii) never screened for cervical cancer during lifetime (yes/no).

Self-perceived health was constructed from three indicators: current general health, current physical health, and current mental health (each indicator had 5 possible answers: very good, good, average, poor, and very poor).

Victimization history was constructed from three indicators based on experiencing the following at least once during one’s lifetime: (i) a violent event (yes/no), (ii) a physical or sexual assault (yes/no) and (iii) an episode of post-traumatic stress disorder (PTSD) according to the MINI (Mini-International Neuropsychiatric Interview) (yes/no) [33].

Social networking opportunities were constructed from three variables: (i) not having been invited to a party or a family celebration during the previous year (yes/no), (ii) not participating in cultural activities (yes/no), and (iii) not going to cafés or restaurants (yes/no).

Caring for children was constructed from four indicators: (i) having at least one child who did not go to school (yes/no), (ii) having at least one child who did not lunch in the school cafeteria (yes/no), (iii) having at least one young child (<3 years old) (yes/no), and (iv) living with at least 3 children (yes/no).

#### Other indicators

We analysed four additional indicators which were directly measured in our dataset: (i) level of education (none, primary, secondary or tertiary), (ii) social health insurance (yes/no), (iii) symptoms of depression, and (iv) official food insecurity status (yes/no). Symptoms of depression were assessed by the CIDI (Composite International Diagnostic Interview) questionnaire [31]. Food insecurity status was assessed using the French version of the Household Food Security Module questionnaire [30,47].

### Statistical analyses

All prevalences and proportions were inversely weighted to each participant’s inclusion probability in accordance with the sampling design [27]. Comparisons between proportions were performed using chi-square tests with a p-threshold < 0.05.

First, we analysed the weighted correlation matrix of observed variables from each latent construct. Second, we performed a scree-plot to check the mono-dimensionality of each latent construct. A scree-plot displays the eigenvalues associated with a component or factor in descending order versus the number of the component or factor. The point where the slope of the curve clearly levels off indicates the number of factors that should be generated.

Third, we estimated measurement models using confirmatory factor analysis (CFA) and finally, tested a structural equation model. The model was assessed with the weighted least squares with mean and variance adjustment (WLSMV) estimator, which is used when models include categorical variables. All estimates were weighted. The coefficients were standardised and ranged from -1 (negative association) to 1 (positive association). The goodness-of-fit of the model was analysed by three evaluation indices: the comparative fit index (CFI), (≥ 0.90) and the Root Mean Square Error of Approximation (RMSEA) (< 0.08).

All analyses were conducted using R software (version 3.2.0), with the "lavaan.survey" packages for estimation of SEM.

## Results

### Population characteristics

Women were 33 years old on average (range: 19–57), 92.7% were born outside France (65.6% in sub-Saharan Africa), 85.1% had at least secondary level education (Table 1). One-third were single. On average, they had two children living with them. Only 20.9% were employed. Their mean monthly household income was 319 €/CU, and 94.2% were living below the poverty line. On average, they had been homeless for approximately 3 years (range: 0–19) and moved between shelters, temporary housing, friends’ houses, and the street three times a year (range: 0–24).

**Table 1:** Characteristics of homeless women* interviewed in the ENFAMS survey in the Greater Paris Area, France, 2013 (N = 656).

Characteristics	Frequency	
	%	_95%_[CI]
**Age**		
Mean(years)	32.6	[32.0–33.2]
Range	19–57	
**Birthplace**		
France	7.3	[4.9–9.7]
Outside of France	92.7	[90.3–95.1]
**Level of education**		
Tertiary	14.8	[11.7–18.0]
Secondary	63.2	[58.7–67.7]
Primary	11.6	[8.8–14.5]
None	10.3	[7.3–13.3]
**Employment**		
Employed	20.4	[16.5–24.2]
Unemployed or student	79.6	[75.8–83.5]
**Income**		
Below poverty line (<908€/cu)	94.2	[91.9–96.6]
Above poverty line (≥908€/cu)	5.8	[3.4–8.1]
**Couple status**		
Living as a couple	64.8	[59.8–69.8]
Not living as a couple	35.2	[30.2–40.2]
**Duration of homelessness**		
Mean (years)	3.2	[2.9–3.5]
Range	0–19	
**Number of children**		
Mean	1.9	[1.8–2.0]
Range	1–9	
**Number of moves per year**		
Mean	2.5	[2.1–2.9]
Range	0–24	

* Women who arrived in France at least one year before the ENFAMS study

### Description of unmet healthcare needs

Of the 656 women in the study sample, 25.1% [21.3–29.0] declared to have at least one unmet healthcare need over the previous year for: financial reasons (57.7%), because they did not have time (18.9%) to go for care, because the care centre was too far away (6.8%), or because they preferred to wait for the condition to go away spontaneously (5.9%) (Table 2). The unmet healthcare needs reported were: consultations with a specialist (45.0%), dental care (42.3%) and consultations with a general practitioner (30.2%). Other unmet healthcare needs concerned: (glasses and contact lenses) (17.0%), laboratory and imaging tests and analyses (10.2%), prescription of drugs (1.2%) and physical therapy (3.2%).

**Table 2:** Reasons and types of unmet healthcare needs of homeless women*, in the ENFAMS survey in the Greater Paris area, France, 2013.

	Frequency	
	%	_95%_[CI]
**Reasons**		
Financial	57.7	[49.0–66.4]
More pressing concerns or not enough time	18.9	[11.7–26.1]
Distance from healthcare centre	6.8	[3.0–10.7]
She preferred to wait for things to improve on their own	5.9	[0–12.3]
Afraid of going for a medical consultation	3.5	[0.5–6.4]
Tired because of pregnancy	3.3	[0.6–6.0]
Did not like doctors or medicine	3.1	[0.5–5.7]
Too difficult or did not know where to go	1.6	[0.2–3.0]
Long waiting times	<1.0	
Hard to make oneself understood in French	<1.0	
**Types**		
Consultations with, visits to, or treatment by a medical specialist	45.0	[36.1–53.9]
Consultations with, visits to, or treatment by a general practitioner	30.2	[21.4–38.9]
Dental prostheses or care	42.3	[33.6–50.9]
Glasses and contact lenses	17.0	[10.9–23.2]
Lab tests, imaging	10.2	[4.2–16.3]
Physical therapy or rehabilitation sessions	3.2	[0.9–5.6]
Prescription of drugs	1.2	[0.1–2.3]
Other	6.8	[2.1–11.4]

* Women who arrived in France at least one year before the ENFAMS study

### Validation of latent constructs

The weighted correlations between the observed variables of each latent construct ranged from 0.2 to 0.7 (details given in supporting information, S1 Table), except for indicators of the “caring for children" latent variable and for the indicator "administrative status" of the latent variable "migration status", which was not retained for the following analysis. All scree-plots checking the mono-dimensionality of each latent variable were satisfactory: all curves levelled off above 1, which indicated that each latent construct was unidimensional (details given in supporting information, S1 Fig).

### Measurement models

The first-order confirmatory factor analysis was estimated with 8 latent variables in the hypothesized model. This model provided a close fit to the data: CFI = 0.853 and RMSEA = 0.046 (CI_95%_[0.042–0.050]), permitting us to not reject the dimensional structure proposed. All factor loadings between each latent variable and its indicators were statistically significant at the 0.05 level.

### Final model

**Fig 2:**
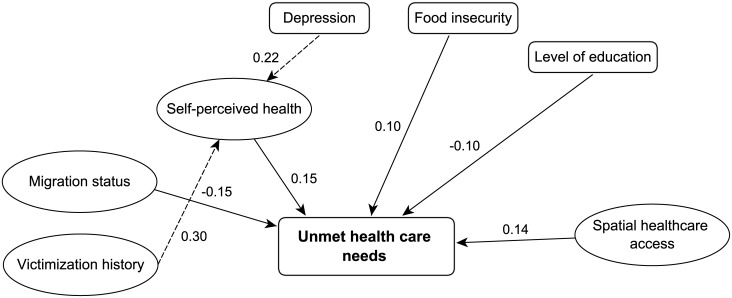
Final model of relationships between latent constructs of the unmet needs of homeless women and their characteristics. Ellipses: latent variables; boxes: observed variables. All coefficients have p values<0.05. Robust indices: CFI = 0.856 and RMSEA = 0.066 (CI95%[0.059–0.072]).

For the final model (Fig 2), CFI = 0.856 and RMSEA = 0.066 (CI_95%_[0.059–0.072]), suggesting an acceptable fit. In the final model, we observed that only two factors had a significant impact on self-perceived health: victimization (β_sd_ = 0.30 CI_95%_[0.18–0.41]) and depression (β_sd_ = 0.22 CI_95%_[0.10–0.34]).

The level of education and the migration status did not have any impact on perceived health but had a direct and significant impact on unmet healthcare needs (β_sd_ = -0.10 CI_95%_[-0.17–-0.03] and β_sd_ = -0.15 CI_95%_[-0.26–-0.04], respectively). Perceived health (β_sd_ = 0.15 CI_95%_[0.05–0.26]), food insecurity (β_sd_ = 0.10 CI_95%_[0.01–0.19]), and spatial healthcare access (β_sd_ = 0.14 CI_95%_[0.02–0.26]) also had a significant and direct impact on unmet healthcare needs.

## Discussion

Our study used data from the first survey performed in a representative sample of homeless families in France. A large set of data was collected in 17 different languages. Our results provide the first ever estimate of unmet healthcare needs in sheltered women with children in France.

### Prevalence of unmet healthcare needs

The prevalence of unmet healthcare needs in homeless women was slightly lower than the 32.3% estimated in the general population in the same area in 2010 [48]. This may seem counterintuitive, given the difficult living conditions of homeless women. Three possible explanations can be put forward for this. First, homeless women may have lower needs perceptions for health, precisely because of their harsh living conditions, or to put it another way, other basic needs, such as food and housing, may compete with their health needs [10,11,49]. Second, they may have lower expectations of the French healthcare system due to poor health literacy. Indeed, many of them may have been not very knowledgeable about the system, because of language barriers or because they emigrated from countries with a different healthcare system. Third, previous research has shown that homeless people generally tend to underreport health issues, to more often pay no attention to worrying symptoms, and to delay going to healthcare services [50]. It must be pointed out however that this fact has been more frequently reported in men.

Finally, our survey population concerned homeless women living in shelters, and excluded those living on the street. Since some studies have shown that the use of healthcare services is influenced by the type of homelessness which people experience, with people who sleep rough having the worst access to care [42], it is possible that we underestimated the prevalence of unmet needs in the total population of homeless women. The precise number of women living in the streets is not known. Although they are becoming increasingly visible in some public spaces in the city of Paris, they remain extremely rare in the Greater Paris area, when compared with the number of those living in shelters (in 2013, the number of sheltered women was: 10 280 _95%_CI[9507–11053]).

### Dimensions with a direct effect on unmet healthcare needs

Many of the determinants of unmet health needs which we hypothesized were confirmed by our analysis. We observed a direct effect of level of education, women’s migration status, spatial healthcare access, a poor self-perceived health, and food insecurity status. Women with a lower educational level, with migration history reported fewer unmet health care needs than the others women. Women with transport difficulties or no satisfaction of neighborhood, with poor self-perceived health or suffered from food insecurity reported higher unmet healthcare needs than the others women. As we mentioned above, migrant women may have fewer unmet needs–independently of their level of education, and independently of perceived health–because of a lack of knowledge of the French healthcare system, and/or because they have low expectations of medicine or medical care, particularly if they emigrated from countries with an underdeveloped or underfunded healthcare system. We need to underline here that we did not observe any significant impact–positive or negative—of migration status on perceived health. This is perhaps because the impact is positive in some migrants (who may have better perceived health, due to the “healthy migrant effect” [51] suggested by theories of selective migration) and negative in others (poor perceived health being brought on by their living conditions in their new country, also called the “exhausted migrant effect” by some authors [52]).

With respect to the level of education, a similar association was observed in different studies on unmet healthcare needs in the French general population [34] and was generally attributed to poor health literacy [45], lack of knowledge of the healthcare system and social rights, and lower expectations of healthcare services [44]. With regard to the direct impact of food insecurity, our competitive hypothesis between basic needs would seem valid: individuals experiencing food insufficiency assign lower priority to healthcare in favor of other basic needs, e.g. housing and food [10,18,53].

Traditional financial barriers (resources and health insurance) were not associated with unmet healthcare needs in our study. Some studies have highlighted that insurance coverage was not sufficient to ensure that the healthcare needs of the homeless are met [13,54]. The absence of any link may be due to two reasons: first, a lack of heterogeneity in the financial situation of the surveyed population (all women were sheltered and their financial characteristics were similar, with a majority of them being unemployed and living below the poverty line [27]). Second, the French welfare system provides specific social health insurance to the poor and/or the undocumented and this may explain why only 12.9% of participants were uninsured at the time of their interview (theoretically, the next time they used a healthcare service, they would automatically be entitled to social health insurance).

As observed in previous studies on homeless people [13,19,41], spatial access to healthcare had an impact on unmet healthcare needs. Our hypothesis that housing instability and frequent moves from one shelter to another may impact this spatial access was not verified. Here too, this may be because of existing opposing relationships: for some women, frequent moves may help to provide a better understanding of the spatial and transportation resources of the Greater Paris area, for others frequent moves contribute to their isolation in unknown environments.

Similarly, the absence of any impact of having children on unmet healthcare needs may be the result of two opposing phenomena. Some studies have indicated that having children may increase mothers’ unmet healthcare needs because they have no one to take care of their children during their absence [13,19], while other studies have shown that having children led to improved access to healthcare (compared with homeless women without children), specifically child healthcare services. The absence of any impact could be also due to the absence of women without children in our study population. Results could have been different if these women had been included in the ENFAMS survey.

In contrast to the French general population [43], social opportunities networking was not associated with a declaration of unmet healthcare needs in this study. We assume that the absence of any significant link here may be due to two reasons. First, there was a lack of heterogeneity of social characteristics: almost all of the women interviewed were socially isolated. In fact, we realized *a posteriori* that some of the questions about social support used in the ENFAMS survey, which had been previously tested on the general population in the SIRS survey, were not adapted to the homeless population which is by definition socially isolated. Second, we can also assume that the homeless women studied do not have a strong or supportive social network (low social influence) because it is constituted by other isolated women like them.

### Dimensions with an indirect effect on unmet healthcare needs through perceived health

Our study suggests that victimization and depression affected perceived health. This result is in line with those from previous studies [29,30,48,49]. Victims of crime are more limited in physical functioning and have a poorer perception of their physical health [55]. In addition, Tan *et al*. showed that women and people from ethnic minority backgrounds (which is similar to our context), tend to suffer disproportionately more from ill-health and may be more vulnerable to victimization [56]. Some studies have also shown that experiencing violent crime was significantly linked to the need to seek medical treatment (hospital visits, medication, visiting a doctor) because of poorer self-perceived health [57]. This may explain the indirect effect (i.e. through perceived health) between victimization and unmet healthcare needs.

### Limitations

Our study had some limitations. First, the ENFAMS survey collected data on homeless women who were sheltered and who have at least one child. Consequently, these results could not be generalizable to all homeless women population in Paris area (women without children and living on the street). Second, unmet healthcare needs and characteristics of homeless women were self-reported. However, Hwang *et al*. indicated that adults experiencing homelessness are quite accurate reporters of their use of healthcare [58]. Third, we did not collect any information about study participants’ knowledge and attitudes regarding medicine, health professionals and the healthcare system, to investigate whether these factors were associated with declarations of unmet healthcare needs [13].

## Conclusions

This study was the first of its kind to examine the relationships between various social and health characteristics and unmet healthcare needs of homeless women with children in France. In the French context, where health insurance coverage and welfare assistance is almost universal, no association was found for traditional factors associated with unmet healthcare needs found in the literature—such as income and health insurance status. This demonstrates that strategies to provide better access to care for this population should not be only financially-based, even if a minimum income and a proper health insurance must be provided to this population. Such strategies should also tackle issues of spatial healthcare access, cultural norms, and perceptions of health. To improve the characterization of the factors for unmet healthcare needs in this population, further analysis is needed of the individual types of need (prevention, dental care, specialists, etc.) and associated reasons (cost, lack of time, lack of knowledge about health services offer, etc.). Furthermore, qualitative studies on women’s attitudes and knowledge toward health and the healthcare system would be of great use, in order to interpret and better disentangle the complex associations and pathways explored in our analysis.

## Supporting information

S1 Table Weighted correlation matrix of observed variables of each latent constructs. (DOCX)

S1 Fig Scree plots of each latent constructs. *Left to right and up to down: scree plot of each latent variables. Financial access to care (Being unemployed, Monthly household income < 211€/CU, Not having received social benefits during the previous year); Migration status (Being born out of France, Time lived in France < ¼ of one’s life, Difficulties in French); Housing history (Duration of homelessness > 24 months, Moves per year > 4, Duration spent in the current shelter < 1 year); Spatial access to healthcare (Difficulties in transport, Not satisfied with public transportation in the neighbourhood, Not satisfied with healthcare providers in the neighbourhood); Healthcare utilization (No consultation a physician during the previous year, No gynaecological follow-up, No papsmear in lifetime); Self-perceived health (Poor or very poor current general health, Poor or very poor current physical health, Poor or very poor current psychological); Victimization history (A violent event, Physical or sexual assault, An episode of PTSD); Social networking opportunities (Not having invited to a party during the previous year, Not participating in cultural activities, Not going to cafés or restaurants).
(EPS)

S1 Dataset Dataset of the ENFAMS survey used in this paper. (CSV)

S1 Listing of Dataset Variables (TXT)
