# Supplementary material for: Unmet healthcare needs in homeless women with children in the Greater Paris area in France
Source: PLoS One. 2017 Sep 6;12(9):e0184138. doi: 10.1371/journal.pone.0184138 (PMC5587267; doi:10.1371/journal.pone.0184138)
Supplement: S1 Table — (DOCX) [file pone.0184138.s001.docx]

S1 Tab. Weighted correlation matrix of observed variables of each latent constructs

| **Financial access to care** | **1** | **2** | **3** |  |
| --- | --- | --- | --- | --- |
| **1.** Being unemployed | 1 |  |  |  |
| **2.** Monthly household income < 211€/CU | 0.34 | 1 |  |  |
| **3.** Not having received social benefits during the previous year | 0.20 | 0.65 | 1 |  |
| **Migration status** | **4** | **5** | **6** | **7** |
| **4.** Being born out of France | 1 |  |  |  |
| **5.** Time lived in France < ¼ of one’s life | 0.46 | 1 |  |  |
| **6.** Difficulties in French | 0.33 | 0.18 | 1 |  |
| **7.** Being undocumented | 0.20 | 0.17 | 0.22 | 1 |
| **Housing history** | **8** | **9** | **10** |  |
| **8.** Duration of homelessness > 24 months | 1 |  |  |  |
| **9.** Moves per year > 4 | -0.46 | 1 |  |  |
| **10.** Duration spent in the current shelter < 1 year | -0.41 | 0.32 | 1 |  |
| **Spatial access to healthcare** | **11** | **12** | **13** |  |
| **11.** Difficulties in transport | 1 |  |  |  |
| **12.** Not satisfied with public transportation in the neighbourhood | 0.28 | 1 |  |  |
| **13.** Not satisfied with healthcare providers in the neighbourhood | 0.24 | 0.50 | 1 |  |
| **Healthcare utilization** | **14** | **15** | **16** |  |
| **14.** No consultation a physician during the previous year | 1 |  |  |  |
| **15.** No gynaecological follow-up | 0.09 | 1 |  |  |
| **16.** No papsmear in lifetime | 0.21 | 0.33 | 1 |  |
| **Self-perceived health** | **17** | **18** | **19** |  |
| **17.** Poor or very poor current general health | 1 |  |  |  |
| **18.** Poor or very poor current physical health | 0.70 | 1 |  |  |
| **19.** Poor or very poor current psychological | 0.52 | 0.52 | 1 |  |
| **Victimization history** | **20** | **21** | **22** |  |
| **20.** A violent event | 1 |  |  |  |
| **21.** Physical or sexual assault | 0.29 | 1 |  |  |
| **22.** An episode of PTSD | 0.64 | 0.27 | 1 |  |
| **Social networking opportunities** | **23** | **24** | **25** |  |
| **23.** Not having invited to a party during the previous year | 1 |  |  |  |
| **24.** Not participating in cultural activities | 0.28 | 1 |  |  |
| **25.** Not going to cafés or restaurants | 0.26 | 0.24 | 1 |  |
| **Caring for children** | **26** | **27** | **28** | **29** |
| **26.** At least one child who did not go to school | 1 |  |  |  |
| **27.** At least one child who did not lunch in the school cafeteria | -0.11 | 1 |  |  |
| **28.** At least one child < 3 years old | 0.06 | 0.43 | 1 |  |
| **29.** Living with at least 3 children | 0.04 | 0.27 | 0.01 | 1 |
